# Galectin-3 Binds to the Allosteric Site and Activates Integrins αvβ3, αIIbβ3, and α5β1, and Lactose Inhibits This Activation

**DOI:** 10.3390/biom16040586

**Published:** 2026-04-15

**Authors:** Yoko K. Takada, Yu-Jui Yvonne Wan, Yoshikazu Takada

**Affiliations:** 1 Department of Dermatology, School of Medicine, University of California Davis, 4645 Second Avenue, Sacramento, CA 95817, USA; 2 Department of Pathology, School of Medicine, University of California Davis, 4645 Second Avenue, Sacramento, CA 95817, USA; yjywan@health.ucdavis.edu; 3 Department of Biochemistry and Molecular Medicine, School of Medicine, University of California Davis, 4645 Second Avenue, Sacramento, CA 95817, USA

**Keywords:** galectin-3, integrin activation, allosteric site, pro-inflammatory action, pro-thrombotic action

## Abstract

Galectin-3 (Gal3) is one of the most pro-inflammatory proteins and a biomarker of inflammatory diseases and cancer. Previous studies showed that Gal3 binds to αv and β1 integrins, but it is unclear how Gal3 binds to integrins. Here, we show that Gal3 bound to soluble αvβ3 and αIIbβ3 integrins in 1 mM Mn^2+^ in cell-free conditions in a glycan-independent manner. Docking simulation predicts that Gal3 binds to the classical RGD-binding site (site 1) of αvβ3, but the predicted Gal3-binding site does not include galactose-binding site. RGDfV or eptifibatide inhibited Gal3 binding to αvβ3 and αIIbβ3, respectively, but lactose, a pan-galectin inhibitor, did not inhibit Gal3 binding to integrins. Point mutations of the predicted site 1 binding interface of Gal3 effectively inhibited Gal3 binding to site 1. Site 2 is involved in pro-inflammatory signaling (e.g., TNF and IL-6 secretion), and we previously showed that pro-inflammatory cytokines (e.g., CCL5 and TNF) bind to site 2 and allosteric integrin activation. Docking simulation predicted that Gal3 binds to site 2 of αvβ3 and α5β1. We found that Gal3 induced allosteric activation of soluble integrins αvβ3, αIIbβ3, and α5β1 in 1 mM Ca^2+^ in cell-free conditions. Point mutations in the predicted site 2 binding interface inhibited Gal3-induced integrin activation, suggesting that Gal3 binding to site 2 is required for Gal3-induced integrin activation. Known anti-inflammatory agents, Ivermectin, NRG1, and FGF1, inhibited integrin activation induced by Gal3 in αvβ3 and αIIbβ3. These findings suggest that Gal3 binding to site 2 may be a potential mechanism of pro-inflammatory and pro-thrombotic action of Gal3.

## 1. Introduction

Integrins are a superfamily of α-β heterodimers of cell adhesion receptors that recognize extracellular matrix (ECM) proteins, cell-surface molecules (e.g., ICAM-1 and VCAM-1), and small proteins (e.g., growth factors such as NRG1, CCL5, FGF2) [1]. We performed virtual screening of a protein data bank (PDB) with the headpiece of integrin αvβ3 as a target using docking simulation. Interestingly, we discovered that multiple pro-inflammatory proteins, including cytokines (e.g., FGF1, FGF2, neuregulin-1, IGF1, IGF2, CD40L, VEGF165, CX3CL1, CXCL12, CCL5) and pro-inflammatory proteins such as sPLA2-IIA and CD62P, bind to the classical ligand-binding site (site 1) [1,2,3,4,5,6,7,8,9,10].

Integrin ligands are known to bind to the classical RGD-binding site in the headpiece (site 1). Integrins are involved in growth factor signaling (integrin–growth factor crosstalk) since antagonists to integrins suppress growth factor signaling [11,12,13]. Previous studies showed that several growth factors induce the integrin–growth factor–cognate growth factor receptor ternary complex on the cell surface. Growth factor mutants defective in integrin binding were defective in signaling functions and act as antagonists of growth factor signaling. These findings suggest that the ternary complex formation is critical for growth factor signaling (ternary complex model). Positions of amino acid residues in site 1 are shown in Supplemental Figure S1a.

Notably, we found that several pro-inflammatory cytokines or pro-inflammatory proteins (e.g., FGF2, CX3CL1, CXCL12, CCL5, CD40L, CD62P, CXCL4, and sPLA2-IIA) bind to the allosteric site (site 2), which is distinct from site 1, and activate integrins in an allosteric manner [7,9,14,15,16,17,18,19,20]. Site 2-derived peptides from integrin β subunits bound to these ligands and suppressed allosteric activation by these ligands. These findings suggest that the binding to these ligands to site 2 is required for allosteric activation of integrins. It has been reported that 25-hydroxycholesterol (25HC), a major inflammatory mediator, binds to site 2, activates integrins and induces inflammatory signals (e.g., secretion of IL-6 and TNF) in macrophages [21]. Notably, ivermectin, a known anti-parasitic and anti-inflammatory small molecule, binds to site 2 and inhibits integrin activation by pro-inflammatory cytokines [22], suggesting that ivermectin is an antagonist of site 2-mediated inflammatory signaling. Also, known anti-inflammatory cytokines NRG1 and FGF1 bind to site 2 and inhibit integrin activation by multiple pro-inflammatory cytokines [20,23]. We propose that site 2 is involved in inflammatory signaling. Positions of amino acid residues in site 2 are shown in Supplemental Figure S1b.

Galectin-3 (Gal3) is a galactoside-binding lectin that is important in numerous biological activities in various organs, including cell proliferation, apoptotic regulation, inflammation, fibrosis, and host defense [24,25]. Gal3 is a diagnostic or prognostic biomarker for certain types of heart disease, kidney disease, viral infection, autoimmune disease, neurodegenerative disorders, and tumor formation. Gal3 positively or negatively affects cancer progression and metastasis [26,27,28]. Gal3 is predominantly in the cytoplasm and expressed on the cell surface and is then often secreted into biological fluids, like serum and urine. It is also released from injured cells and inflammatory cells under various pathological conditions. The mechanism of pro-inflammatory action of Gal3 is unclear. Gal3 has been reported to interact with β1 integrins (α2β1, α3β1). Also, Gal3 enhances cell adhesion to ECM [29], binds to αv integrins [30] and α5β1 integrin [31]. We hypothesized that Gal3 activates integrins and induces pro-inflammatory signals by binding to site 2, like pro-inflammatory cytokines.

It has been proposed that Gal3 binding to integrins is mediated by carbohydrate binding. We previously discovered that the lectin domain of P-selectin (CD62P), another carbohydrate-binding protein, binds to integrins through protein–protein interaction [9]. P-selectin lectin domain binds to site 1 and site 2 and activates integrins through binding to site 2. We showed that Gal3 binds to site 1 of integrins in a glycan-independent manner and RGD-dependent manner. We also showed that Gal3 binds to site 2 and allosterically activates integrins as several pro-inflammatory cytokines. We propose that Gal3 induces pro-inflammatory signals by binding to site 2.

## 2. Materials and Methods

### 2.1. Materials

Ivermectin (IVM) was obtained from eMolecules (San Diego, CA, USA). We used stock IVM (10 mM) in 95% ethanol and diluted it in water. Human soluble αvβ3 (IT3-H52E3) and αIIbβ3 (IT3-H52W8) were obtained from ThermoFischer (Waltham, MA, USA). Mab AV10 (to human β3) was kindly provided by Brunie Felding (Scripps Research Institute, La Jolla, CA, USA). HRP-conjugated anti-His tag (C-terminal) antibody was purchased from Qiagen (Valencia, CA, USA).

Galectin-3 (Gal3): cDNA encoding the carbohydrate-binding domain of Gal3 (residues 114–250) [LIVPYNLPLPGGVVPRMLITILGTVKPNANRIALDFQRGNDVAFHFNPRFNENNRRVIVCNTKLDNNWGREERQSVFPFESGKPFKIQVLVEPDHFKVAVNDAHLLQYNHRVKKLNEISKLGISGDIDLTSASYTMI] were synthesized and subcloned into the BamHI/EcoRI site of pET28a vector. Protein synthesis was induced by IPTG in *E. coli* BL21. Protein was synthesized as insoluble inclusion bodies and purified in denaturing conditions (8 M urea) in Ni-NTA Sepharose and refolded as previously described [2].

Cyclic β3-site 2 peptide: We synthesized the 29 mer cyclic β3 site 2 peptide C260—RLAGIV[QPNDGSHVGSDNHYSASTTM]C288 (C273 is changed to S273) as previously described [23].

β3 SDL peptide: The cDNA fragment encoding the specificity-determining loop (SDL) (residues 158–188 of β3 [DKPVSPYMYISPPEALENPCYDMKTTCLPMF]) was synthesized as previously described [23]. Proteins were synthesized in *E. coli* BL21 and purified using Glutathione Sepharose affinity chromatography.

### 2.2. Methods

Docking simulation: Docking simulation between the C-terminal region of galectin-3 and integrin αvβ3 or α5β1 was performed as described [4]. The ligand was presently compiled to a maximum size of 1024 atoms. Atomic solvation parameters and fractional volumes were assigned to the protein atoms by using the AddSol utility, and grid maps were calculated by using the AutoGrid utility in AutoDock 3.05. A grid map with 127 × 127 × 127 points and a grid point spacing of 0.603 Å included the headpiece of αvβ3 or α5β1. Kollman ‘united-atom’ charges were used. AutoDock 3.05 uses a Lamarckian genetic algorithm (LGA) that couples a typical Darwinian genetic algorithm for global searching with the Solis and Wets algorithm for local searching. The LGA parameters were defined as follows: the initial population of random individuals had a size of 50 individuals; each docking was terminated with a maximum number of 1 × 10^6^ energy evaluations or a maximum number of 27,000 generations, whichever came first; and mutation and crossover rates were set at 0.02 and 0.80, respectively. An elitism value of 1 was applied, which ensured that the top-ranked individual in the population always survived into the next generation. A maximum of 300 iterations per local search were used. The probability of performing a local search on an individual was 0.06, whereas the maximum number of consecutive successes or failures before doubling or halving the search step size was 4. Clustering analysis was performed using Autodocktools.

ELISA-type binding assay: Wells of a 96-well microtiter plate were incubated with Gal3 in PBS for 1 h at room temperature, and the remaining binding sites were blocked by incubating with 0.1% heat-treated BSA (80 °C for 10 min). Wells were incubated with recombinant soluble αvβ3 or αIIbβ3 (1 µg/mL) in HEPES-Tyrodes buffer with 1 mM Mn^2+^ for 1 h at room temperature. After washing the wells, bound integrins were quantified using anti-β3 mAb (AV10) and HRP-conjugated anti-GST and peroxidase substrate.

ELISA-type activation assay: Wells of a 96-well microtiter plate were incubated with recombinant fibrinogen fragment γC399tr (specific ligand for αvβ3) or γC390-411 (specific to αIIbβ3) (50 μg/mL in PBS) for 1 h at room temperature. Remaining binding sites were blocked by incubating with 0.1% heat-treated BSA (80 °C for 10 min). Wells were incubated with recombinant soluble αvβ3 or αIIbβ3 (1 μg/mL) in the presence of Gal3 in HEPES-Tyrodes buffer with 1 mM Ca^2+^ for 1 h at room temperature. After washing unbound integrins, bound integrins were quantified using anti-β3 mouse Ab (AV10) and HRP-conjugated anti-mouse IgG and peroxidase substrate.

Flow cytometry: Fibronectin type III domains 8–11 (FN8-11) specific to α5β1 fused to GST [32] were synthesized as described in the cited references. CHO cells were cultured in Dulbecco’s modified Eagle’s medium (DMEM)/10% fetal calf serum. The cells were resuspended with HEPES-Tyrodes buffer/0.02% BSA (heat-treated at 80 °C for 20 min to remove contaminating cell adhesion molecules). CHO cells were then incubated with Gal3 for 30 min on ice and then incubated with FITC-labeled FN8-11) for 30 min at room temperature. The cells were washed with PBS/0.02% BSA and analyzed by BD Accuri flow cytometer (Becton Dickinson, Mountain View, CA, USA). The data were analyzed using FlowJo 7.6.5.

Site-directed mutagenesis was performed using the QuickChange method [33].

Statistical Analysis: We used Prism 10 (Graphpad Software, Boston, MA, USA) to test treatment differences using ANOVA and Tukey multiple comparison tests to control the global type I error.

## 3. Results

### 3.1. Gal3 Binds to Soluble αvβ3 and αIIbβ3 in 1 mM Mn^2+^ in an RGD-Dependent and Glycan-Independent Manner

**Figure 1:**
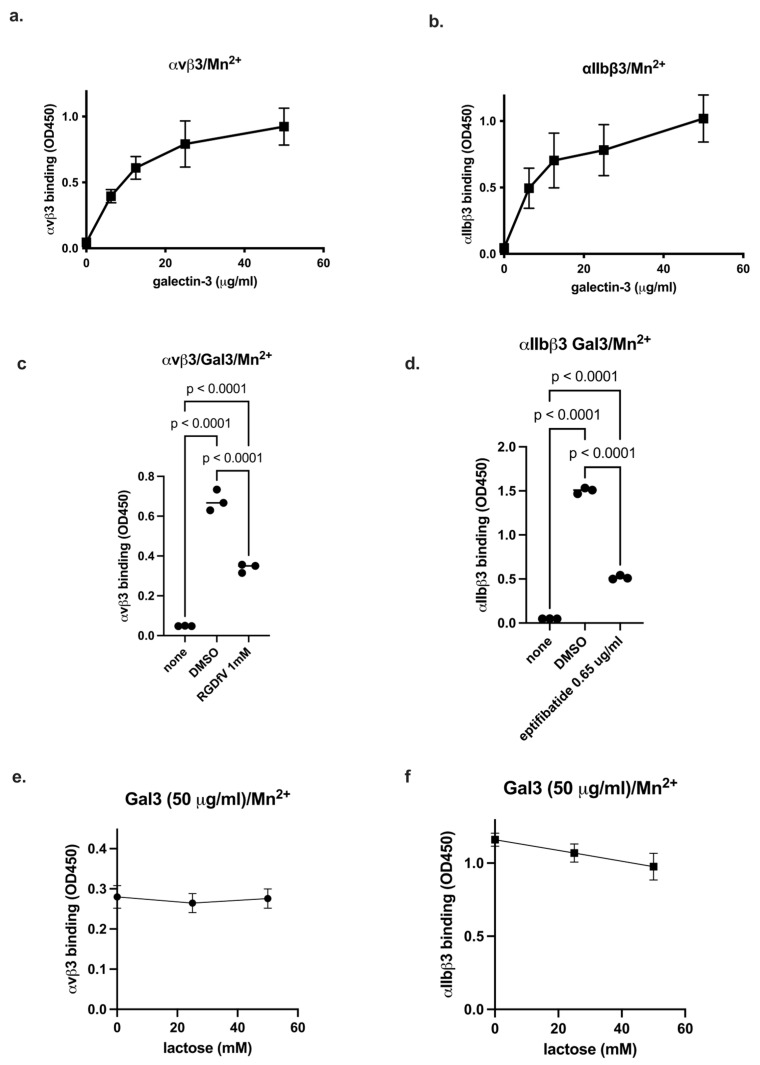
Gal3 binding to soluble integrins αvβ3 and αIIbβ3 in 1 mM Mn^2+^. (**a**,**b**) Gal3 binding to soluble integrins was measured as described in Methods. Briefly, Gal3 was immobilized to wells and incubated with soluble αvβ3 or αIIbβ3. Bound integrins were measured using anti-β3 and HRP-conjugated anti-mouse IgG. Data is shown as means +/− SD of triplicate experiments. (**c**,**d**) Effect of integrin inhibitors RGDfV (1 mM) and eptifibatide (0.65 μg/mL) on Gal3 binding to soluble αvβ3 and αIIbβ3, respectively, was measured. (**e**,**f**) Effect of lactose (up to 50 mM) on Gal3 binding to soluble αvβ3 and αIIbβ3, respectively, was measured. There was no significant effect of lactose on Gal3 binding to integrins. Data is shown as means +/− SD of triplicate experiments.

Binding of Gal3 to integrins was studied in ELISA-type binding assay using soluble integrins (ectodomain) in 1 mM Mn^2+^, which fully activate integrins. We found that αvβ3 and αIIbβ3 bound to immobilized Gal3 in a dose-dependent manner (Figure 1a,b). RGDfV, a specific inhibitor of αvβ3, inhibited the binding of soluble αvβ3 to immobilized Gal3 in 1 mM Mn^2+^ at 1 mM (Figure 1c). Eptifibatide, a specific inhibitor for αIIbβ3, inhibited at 0.65 μg/mL the binding of soluble αIIbβ3 to Gal3 (Figure 1d). These findings suggest that Gal3 binds to αvβ3 and αIIbβ3 in an RGD-dependent manner, although Gal3 has no RGD motif. Lactose, a pan-galectin inhibitor, did not significantly affect Gal3 binding to αvβ3 and αIIbβ3 (up to 50 mM) (Figure 1e,f), suggesting that Gal3 bind to integrins in a glycan-independent manner.

### 3.2. Docking Simulation Predicts That Gal3 Binds to the Classical RGD-Binding Site (Site 1) of Integrin αvβ3

**Figure 2:**
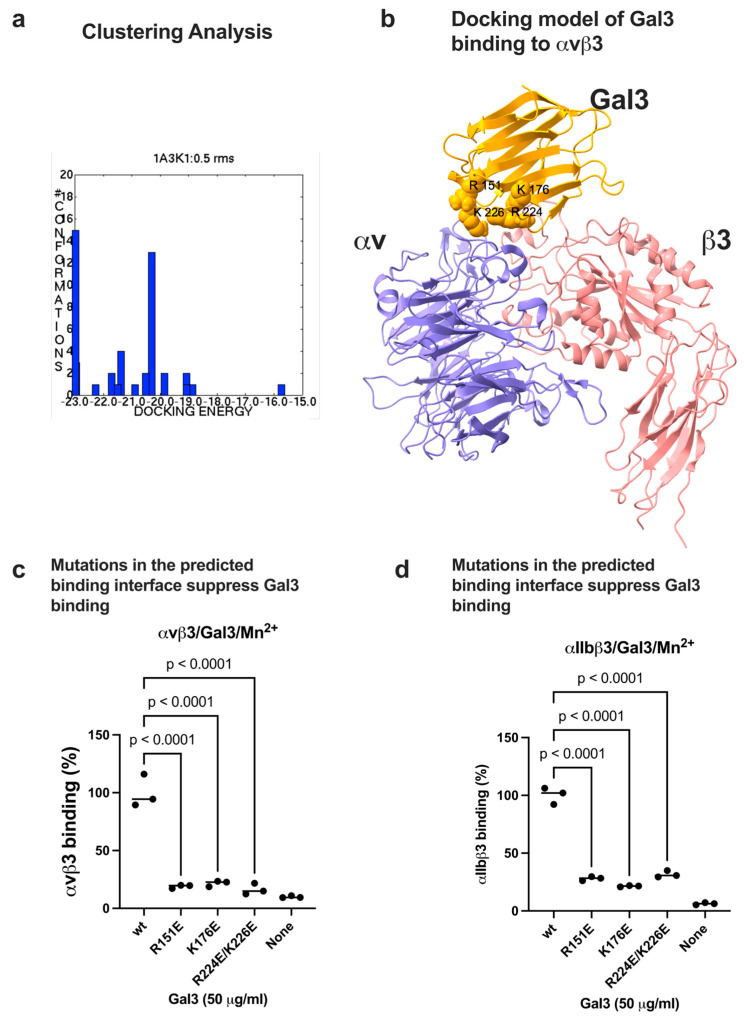
Docking simulation of Gal3 binding to αvβ3. Docking simulation between Gal3 and open headpiece αvβ3 (1L5G.pdb) was performed using autodock3 as described in the Section 2. (**a**) Clustering analysis of docked poses. The first cluster with docking energy −23 kcal/mol represents the most likely poses when Gal3 binds to site 1. (**b**) Docking model of Gal3 binding to site 1 of αvβ3 (1L5G.pdb). (**c**) Binding of Gal3 mutations to αvβ3 in 1 mM Mn^2+^. Point mutations of amino acid residues of Gal3 in the predicted binding interface effectively suppressed Gal3 binding to soluble αvβ3. (**d**) Binding of Gal3 mutations to αIIbβ3 in 1 mM Mn^2+^. Point mutations of amino acid residues of Gal3 in the predicted binding interface effectively suppressed Gal3 binding to soluble αIIbβ3. Data is shown as means +/− SD of triplicate experiments.

We performed docking simulation between Gal3 (1A3K.pdb) and the integrin αvβ3 headpiece (open headpiece conformation, 1L5G.pdb). The 3D structures of open- and closed-headpiece forms of integrin were well-defined in αvβ3 (1L5G and 1JV2.pdb, respectively). Therefore, docking models of only αvβ3 are shown. Carbohydrate molecules were removed from the αvβ3 headpiece or Gal3. The simulation predicts that Gal3 binds to the classical ligand (RGD) binding site (site 1) of αvβ3 (docking poses are clustered at docking energy 23 kcal/mol) (Figure 2a). Consistently, several amino acid residues involved in RGDfV binding in 1L5G.pdb are involved in Gal3 binding (Table 1). Predicted amino acid residues involved in Gal3-αvβ3 interaction are shown in Table 1. To prove if the docking model is correct, we introduced mutations of basic residues (Arg151, Lys176, and Arg224/Arg226) to Glu (charge reversal mutation) in the predicted integrin binding interface (Figure 2b). We found that R151E, K176E, and R224E/R226E mutations effectively suppressed the binding of integrins αvβ3 and αIIbβ3 to Gal3 in ELISA-type binding assays (Figure 2c,d), suggesting that these amino acid residues are critical for binding to site 1 in these integrins. These findings are consistent with the docking model. Several Gal3 residues are close to galactose in 3D structures of the Gal3-galactose complex (His158, Asn160, Arg162, Val172, Cys173, Thr175, Asn174, Trp181, Gly182, Glu184, and Glu185 in 1A3K.pdb) (the carbohydrate-binding domain, CRD) but are not in the Gal3-site 1 interface (Table 1), consistent with the idea that galactose-Gal3 interaction may not be involved in Gal3 binding to site 1.

### 3.3. Gal3 Binds to Site 2 and Induces Allosteric Integrin Activation

**Figure 3:**
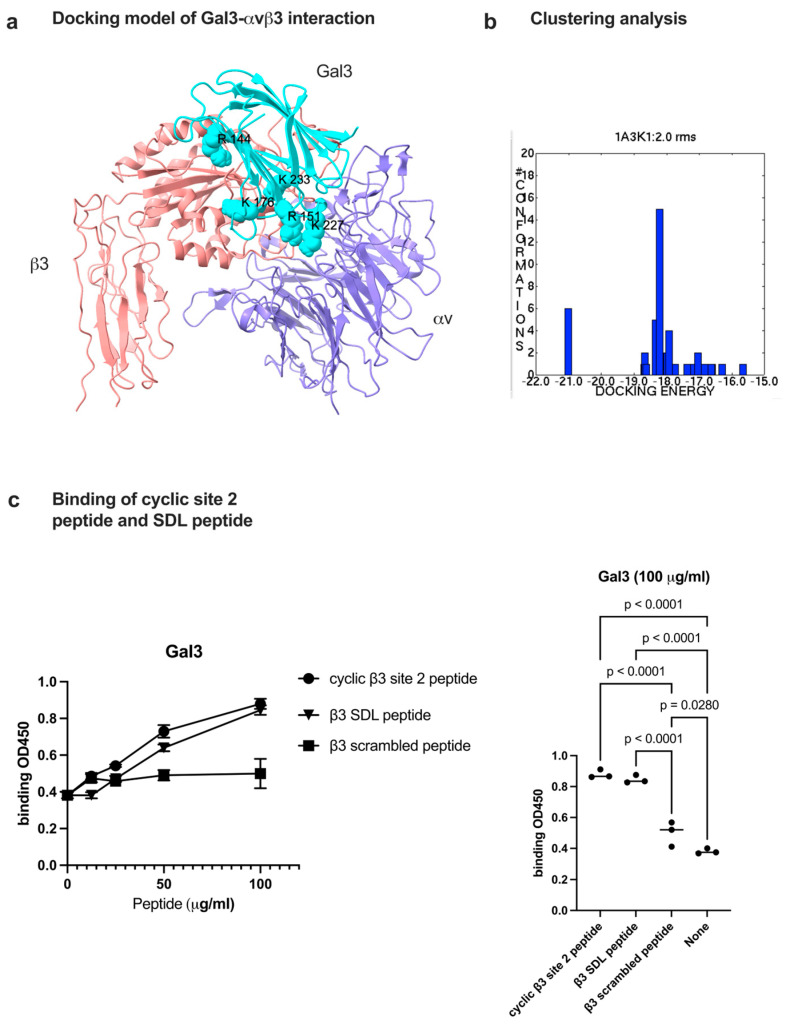
Docking simulation of galectin-3 to site 2 of αvβ3 (1JV2.pdb). Docking simulation between Gal3 and closed-headpiece αvβ3 (1JV2) was performed using autodock3 as described in the Section 2. (**a**) Docking model of galectin-3-αvβ3 interaction. The positions of amino acid residues in the predicted site 2 binding site. Amino acid residues involved in the interaction are shown in Table 2. (**b**) The clustering analysis of docked poses. The first cluster with docking energy −21 kcal/mol represents the most likely poses when Gal3 binds to site 2. (**c**) Binding of cyclic site 2 peptide and β3 SDL peptide to Gal3. Peptide binding was performed as described in the Section 2. Cyclic site 2 peptide, β3 SDL peptide and β3 SDL peptide bind significantly better to Gal3 than scramble peptide. Data is shown as means +/− SD of triplicate experiments.

Previous studies showed that several pro-inflammatory cytokines (e.g., CX3CL1, CXCL12, CCL5, and CD40L) bind to site 2 of integrins and induce allosteric integrin activation [7,14,17,18]. The binding of 25-hydroxycholesterol has been shown to induce pro-inflammatory signaling (e.g., secretion of IL-6 and TNF) [21]. Gal3 enhances platelet aggregation and thrombosis [34] possibly through activation of platelet αIIbβ3. We thus hypothesized that Gal3 binds to site 2 and allosterically activates integrins αvβ3 and αIIbβ3, leading to pro-inflammatory signaling and platelet activation. To prove this hypothesis, we performed docking simulation between Gal3 (1A3K.pdb) and integrin αvβ3 headpiece (inactive, closed-headpiece form, 1JV2.pdb). The simulation predicts that Gal3 binds to the allosteric ligand binding site of αvβ3 (site 2) (Figure 3a). The docking poses with the highest affinity (docking energy −21 kcal/mol) are clustered in cluster 1 (Figure 3b), which represent the likely poses that binds to site 2. Previous studies showed that ligands that bind to site 2 also bind to site 2 peptides and/or SDL (specificity-determining loop) peptides (Table 2) [23]. We found that Gal3 binds to cyclic site 2 peptides and SDL peptides from the β3 subunit (Figure 3c). These findings suggest that Gal3 binds to site 2.

**Figure 4:**
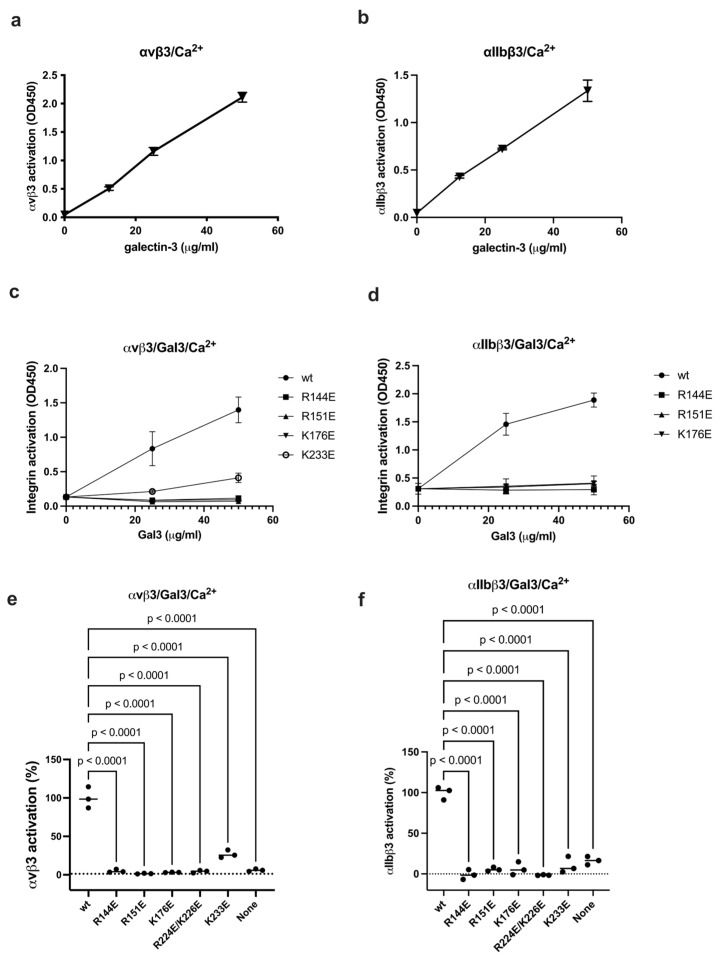
Gal3 binds to the allosteric binding site (site 2) and induces allosteric activation of soluble integrins. (**a**,**b**) Activation of soluble integrins. Gal3 was incubated with soluble αvβ3 (**a**) or αIIbβ3 (**b**) in 1 mM Ca^2+^. Bound integrins were measured using anti-human β3 and HRP-conjugated anti-mouse IgG. (**c**–**f**). Mutations of the site 2 binding interface of Gal3 inhibit activation of soluble integrin αvβ3 (**c**) and soluble αIIbβ3 (**d**). (**e**,**f**) Integrin activation by Gal3 mutations (at 50 μg/mL). Data is shown as means +/− SD of triplicate experiments.

Binding of Gal3 to site 2 was studied in ELISA-type activation assays. Wells were coated with fibrinogen fragments (γC399tr for αvβ3, and γC390-411 for αIIbβ3) and incubated with soluble αvβ3 or αIIbβ3 in the presence of Gal3, and bound integrins were quantified. We found that Gal3 enhance binding of αvβ3 and αIIbβ3 to immobilized fibrinogen fragments in a dose-dependent manner (Figure 4a,b), suggesting that Gal3 activates these integrins. Amino acid residues involved in Gal3-αvβ3 interaction are shown in Table 2. We noticed that the site 2 binding interface of Gal3 overlaps with that of the site 1 binding interface. To study if the docking model is correct, we studied if mutations of several basic residues (Arg144, Arg151, Lys176, Arg224/Lys226, Lys233) to Glu in the αvβ3-binding interface of Gal3 affect integrin activation. These mutants effectively suppressed the ability of Gal3 to activate sol αvβ3 and αIIbβ3 (Figure 4c–f), suggesting that these amino acid residues are involved in site 2 binding, which is consistent with the docking model.

### 3.4. Gal3 Binds to Site 2 and Induces Allosteric Activation of Soluble α5β1 Integrin

**Figure 5:**
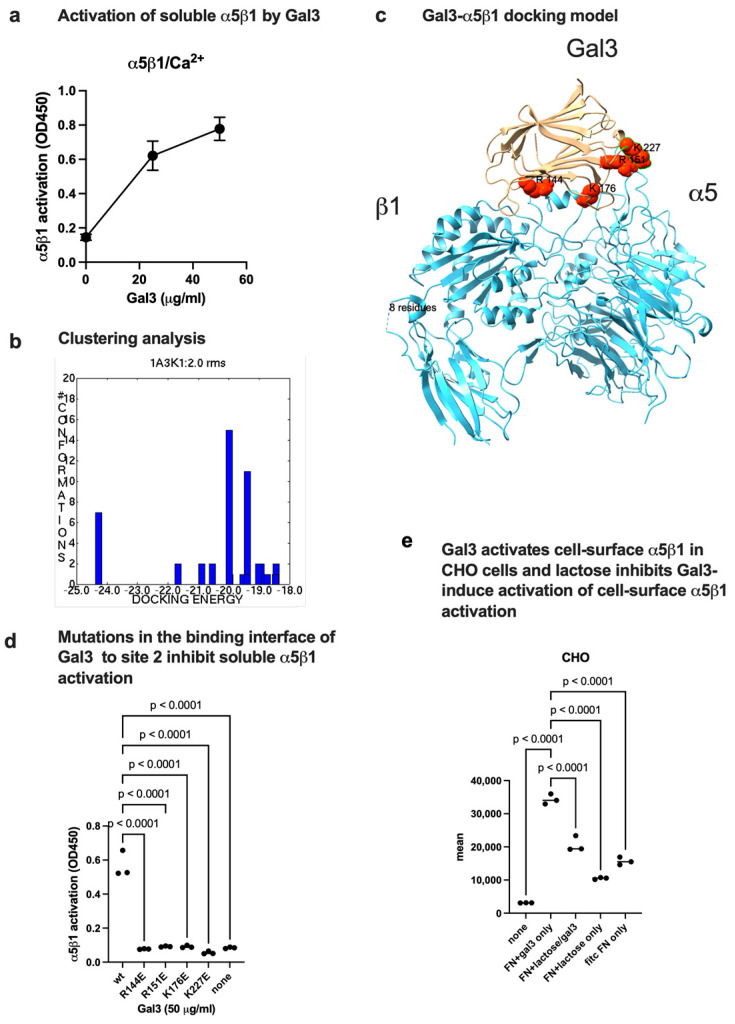
Gal3 activates α5β1 by binding to site 2. Docking simulation of α5β1-Gal3 interaction. Docking simulation of α5β1 (4wjk.pdb) and Gal3 (1A3K.pdb) was carried out as described in the Section 2. (**a**) Activation of soluble α5β1 by Gal3 in 1 mM Ca^2+^ in a dose-dependent manner. α5β1 activation was assayed as described for αvβ3 except that His-tagged soluble α5β1 was used. α5β1 was quantified using anti-His antibody conjugated with HRP. (**b**) Clustering analysis. The first cluster with docking energy −24.26 Kcal/mol represents the poses that bind to site 2 and activate α5β1. Docking simulation was performed as described in the Section 2. (**c**) Docking model of Gal3 binding to α5β1 in the first cluster. (**d**) Effect of mutations in the predicted site 2 binding interface of Gal3 to α5β1. Data is shown as means +/− SD of triplicate experiments. (**e**) Activation of cell-surface integrin α5β1 by Gal3. CHO cells (α5β1+) were incubated with Gal3 (50 μg/mL) and/or lactose (50 mM) in 1 mM Ca^2+^ and FITC FN8-11. Bound FN8-11 was measured in flow cytometry. Data is shown as mean fluorescent intensity +/− SD of triplicate experiments.

Previous studies showed that Gal3 binds to purified transmembrane *α*5*β*1 integrin in a microcavity-suspended lipid bilayer (MSLB) platform of complex lipid composition in an activating cation (Mn^2+^) [31]. These findings suggest that Gal3 binds to site 1 of *α*5*β*1. It is unclear if α5β1 is activated by Gal3 by binding to site 2. We found that wt Gal3 activated soluble α5β1 (ectodomain) in 1 mM Ca^2+^ in activation assays using biotinylated soluble α5β1 (Figure 5a). Docking simulation predicts that Gal3 binds to site 2 of α5β1 at comparable affinity to that of αvβ3 site 2 (Figure 5b). Docking model shows that Gal3 binds to site 2 of α5β1 (Figure 5c). Predicted amino acid residues of Gal3 involved in α5β1 binding are shown in Table 3. Interestingly, several Gal3 mutants (R144E, R151E, K176E, and K227E) in the site 2 binding interface inhibited α5β1 activation by Gal3 (Figure 5d), suggesting that α5β1, αvβ3, and αIIbβ3 bind to site 2 in a similar manner. These findings suggest that Gal3-induced allosteric activation is not limited to β3 integrins.

### 3.5. Activation of Cell-Surface Integrin α5β1 by Gal3

It is unclear if Gal3 activates cell-surface integrins. To address this question, we incubated CHO cells that express α5β1 with Gal3 and FITC-labeled fibronectin fragment (FN8-11) specific to integrin α5β1 in 1 mM Ca^2+^. Ligand binding was measured in flow cytometry. Gal3 enhanced ligand binding to α5β1, suggesting that Gal3 induced activation of α5β1 integrin and lactose inhibited Gal3-induced activation of cell-surface α5β1 (Figure 5e). These findings suggest that Gal3 and lactose similarly affect soluble and cell-surface α5β1.

### 3.6. Anti-Inflammatory FGF1, NRG1, and IVM Inhibited Gal3-Induced Integrin Activation

**Figure 6:**
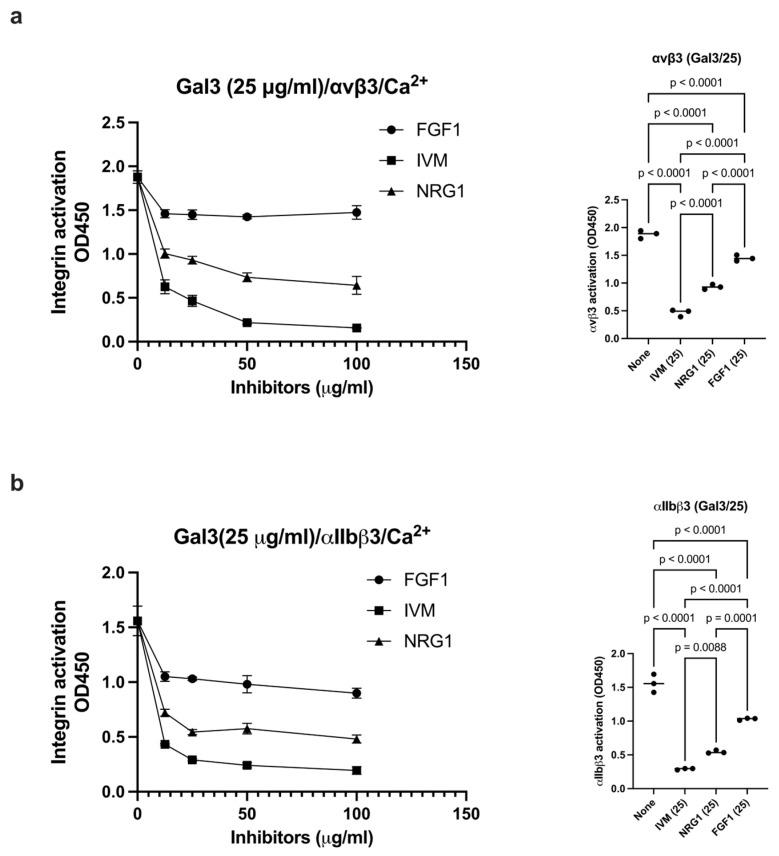
Anti-inflammatory FGF1, NRG1, and IVM suppress Gal3-induced integrin activation of αvβ3 (**a**) and αIIbβ3 (**b**). Activation of soluble αvβ3 (**a**) or αIIbβ3 (**b**) by Gal3 (25 μg/mL) in 1 mM Ca^2+^ was measured as described in the Section 2. The effect of FGF1, IVM, or NRG1 (up to 100 μg/mL) was studied. Data is shown as means +/− SD of triplicate experiments. The statistical analysis of the effect of FGF1, NRG1, and IVM at 25 μg/mL was performed.

Previous studies showed that anti-inflammatory FGF1 binds to site 2 but does not activate integrins. Instead, FGF1 inhibited integrin activation by pro-inflammatory FGF2 [20]. Also, anti-inflammatory NRG1 inhibited allosteric integrin activation induced by multiple pro-inflammatory cytokines (e.g., CCL5) [23]. We also discovered that anti-inflammatory IVM, an anti-parasite agent, binds to the center of site 2 and inhibits integrin activation induced by multiple inflammatory cytokines (e.g., TNF, CCL5, CXCL12) [22], suggesting that IVM is a site 2 antagonist. We hypothesized that IVM may inhibit integrin activation by Gal3. We found that IVM, NRG1 and FGF1 (in this order) inhibited integrin activation induced by Gal-3 (Figure 6a,b), suggesting that these anti-inflammatory agents act as antagonists for Gal-3 induced inflammatory signaling.

## 4. Discussion

In the present study, we first establish that Gal3 binds to the classical RGD-binding site (site 1) of soluble integrin αvβ3 and αIIbβ3. RGDfV inhibited Gal3 binding to αvβ3 and eptifibatide inhibited Gal3 binding to αIIbβ3 in 1 mM Mn^2+^, suggesting that Gal3 binds to site 1 in an RGD-dependent manner. Notably, this binding of Gal3 to integrins was not affected by lactose, suggesting that glycans are not involved in site 1 binding. Notably, the integrin-binding interface of Gal3 does not include known carbohydrate-binding sites, which is consistent with the idea that Gal3-integrin binding is not dependent on glycan binding. Docking simulation predicts that Gal3 binds to site 1 of αvβ3. Point mutations in the predicted site 1 binding interface of Gal3 effectively suppressed Gal3 binding to site 1 of soluble integrin αvβ3 and αIIbβ3, consistent with the docking model. Also, these findings suggest that Gal3 is like a known integrin ligand (e.g., ECM ligands). Gal3 is known to be one of the most pro-inflammatory proteins. Previous studies showed several inflammatory cytokines (e.g., CCL5, TNF, CX3CL1, CXCL12, sPLA2-IIA) that bind to site 2 and allosterically activate integrins αvβ3 and αIIbβ3. We showed that Gal3 binds to site 2 and acts like pro-inflammatory cytokines. Docking simulation predicts that Gal3 binds to site 2 of αvβ3, αIIbβ3 and α5β1. Consistently, Gal3 activated these integrins in 1 mM Ca^2+^. Point mutations in the predicted site 2 binding interface suppressed Gal3-induced activation of these integrins, suggesting that Gal3 binding to site 2 is required for Gal3-induced integrin activation. Gal3 bound to cyclic site 2 peptides and the SDL peptide from the integrin β3 subunit, consistent with the idea that Gal3 binding to site 2 is required for Gal3-induced integrin activation. Notably, site 2-binding interfaces of Gal3 overlap when Gal3 binds to site 2 of three different integrins, suggesting that a single Gal3 antagonist to the site 2 binding site may effectively suppress pro-inflammatory action of Gal3. Gal3 is known to enhance platelet aggregation and thrombosis [34]. The present study provides evidence that Gal3 binds to site 2 of αIIbβ3 and that this interaction may result in platelet activation and aggregation, leading to pro-thrombotic action of Gal3. Furthermore, FGF1 [20], ivermectin [22] and NRG1 [23] inhibited Gal3-induced integrin activation, suggesting that these known anti-inflammatory cytokines or compounds block Gal3 binding to site 2 and inhibit Gal3-induced pro-inflammatory and pro-thrombotic actions. It should be noted that pro-inflammatory cytokines (e.g., TNF and CCL5) and pro-inflammatory proteins (e.g., Gal3, sPLA2-IIA, CD62P) bind to site 2 and allosterically activate integrins independent of outside-in signaling. Also, anti-inflammatory cytokines (e.g., FGF1 and NRG1) and potential anti-inflammatory compounds (ivermectin) bind to site 2 and inhibit allosteric integrin activation. These findings suggest that site 2 is a potential therapeutic target and site 2-mediated integrin activation is a potential marker for inflammation and thrombosis.

## 5. Conclusions

In the present study, docking simulation of interaction between Gal3 (CRD) and integrin αvβ3 predicted that Gal3 binds to site 1. Gal3 specifically binds to site 1 of soluble integrins αvβ3 and αIIbβ3 in cell-free conditions in 1 mM Mn^2+^. Lactose did not affect galectin binding to site 1, but integrin inhibitors did, suggesting that carbohydrates may not play a critical role in integrin binding. Mutations in the predicted binding interface to site 1 inhibited the binding. Furthermore, docking simulation predicted that Gal3 binds to site 2. Gal3 potently activated soluble β3 integrins in cell-free conditions in 1 mM Ca^2+^, suggesting that Gal3 induces integrin activation in an allosteric manner. Cyclic site 2-derived peptides bound to Gal3, which is consistent with the prediction that Gal3 binds to site 2. Point mutations in the predicted site 2 binding interface effectively suppressed Gal3-induced integrin activation. In contrast to site 1, lactose potently inhibited Gal3-induced integrin activation. Notably, ivermectin, NRG1 and FGF1 inhibited integrin activation induced by Gal3, suggesting that Gal3 binding to site 2 is involved in integrin activation by Gal3. Allosteric activation of αIIbβ3 by Gal3 may trigger platelet aggregation and thrombosis. Allosteric activation of αvβ3 may trigger inflammatory signals through site 2 and induce inflammatory signals. We propose that Gal3 binding to site 2 may be critically involved in pro-inflammatory and pro-thrombotic signals by Gal3 and that lactose, ivermectin, NRG1 and FGF1 have potential as antagonists for Gal3-mediated diseases.

## Figures and Tables

**Table 1:** Predicted amino acid residues that are involved in Gal3-integrin αvβ3 binding. Amino acid residues within 0.6 nm between Gα13 and IGF1R kinase were selected using Pdb Viewer (version 4.1). Amino acid residues of Gal3 selected for mutagenesis are in bold. Amino acid residues involved in RGDfV binding in 1L5G.pdb are underlined.

1A3K (−23kcal/mol)	αv	β3 (1L5G)
**Arg151**, Asn153, Asp154, Asn166, Asn174, Thr175, **Lys176**, Leu177, Asp178, Asn179, Asn180, Trp181, Gly182, Arg183, Glu184, Glu185, Arg186, **Arg224, Lys226**, Lys227, Glu230	Ala149, Asp150, Tyr178, Trp179, Ala215, Ile216, Asp218, Asp219, Aeg248,	Tyr166, Asp179, Met180, Lys181, Thr182, Arg214, Asn215, Arg216, Asp217, Ala218, Pro219, Glu220, Asp251, Ala252, Lys253, Thr311, Glu312, Asn313, Val314, Asn316, Leu333, Ser334, Met335, Asp336, Ser337

**Table 2:** Predicted amino acid residues of Gal3 binding to site 2 of αvβ3.

1A3K (−21 kcal/mol)	αv (1JV2.pdb)	β3
Leu114, Ile115, Val116, Pro117, Tyr118, Asn119, Leu120, Pro121, Pro123, Gly124, Gly125, **Arg144**, Ala146, Asp148, Gln150, **Arg151**, Gly152, Asn153, Asp154, Val155, His158, **Lys176**, Asn179, Trp181, **Lys227**, Asn229, Glu230, Ser232, **Lys233**, Leu234, Gly235, Ile236, Ser237, Ala245, Ser246, Tyr247, Thr248,	Glu15, Gly16, Lys42, Asn44, Val51, Glu52, His91, Arg122, Ser399	**Pro160, Met165, Ile167, Ser168, Glu171, Glu174, Asn175, Leu185, Pro186, Met187, Phe188,** Lys191, His192, Val193, Lys209, Gln210, Ser211, Cys273, His274, Val275, Gly276, Ser277, Asp278, Asn279, His280, Tyr281, Ser282, Ala283, Ser284, Thr285, Thr285

Amino acid residues within 0.6 nm between galectin-3 and αvβ3 were selected using Pdb Viewer (version 4.1). Amino acid residues of Gal3 selected for mutation are shown in bold. Amino acid residues in the site 2 peptide are underlined. Amino acid residues in SDL peptide are in red.

**Table 3:** Predicted amino acid residues involved in Gal3 and α5β1 site2 binding.

1A3K (−24.28 kcal/mol)	α5 (4WJK.pdb)	β1
Ile115, Val116, Pro117, Leu122, Gly124, Gly125, Lys139, Asn141, Asn143, **Arg144**, Ala146, Asp148, Gln150, **Arg151**, Gly152, Asn153, Asp154, Val155, Asn164, Glu165, Asn166, Asn167, **Arg168**, **Lys176**, Leu177, Asp178, Asn179, Asn180, Trp181, **Lys227**, Asn229, Glu230, Leu231, Ser232, **Lys233**, Gly235, Ile236, Ser237, Gly238, Asp239,	Pro47, Gly48, Val49, Leu50, Ser77, Ser87, Glu88, Gly89, Glu90, Glu91, Pro92, Glu94, Tyr97, Leu98, Ser120, Ser129	Met173, Thr178, Thr179, Pro180, Leu183, Arg184, Asn185, Ser189, Gln191, Asn192, Thr194, Thr195, Lys200, Glu214, Lys218, Arg220, Glu284, Asn285, Asn286, Pro323

Amino acid residues within 0.6 nm between galectin-3 and α5β1 were selected using Pdb Viewer (version 4.1). Amino acid residues of Gal3 selected for mutation are shown in bold.

## Data Availability

We will share existing datasets or raw data that have been analyzed in the manuscript upon request.

## Supplementary Materials

The following supporting information can be downloaded at: https://www.mdpi.com/article/10.3390/biom16040586/s1, Figure S1: title. Positions of amino acid residues involved in the classical RGD-binding site (site 1) and the allosteric site (site 2).
