# Supplementary figures and images for: Galectin-3 Binds to the Allosteric Site and Activates Integrins αvβ3, αIIbβ3, and α5β1, and Lactose Inhibits This Activation
Source: Biomolecules. 2026 Apr 15;16(4):586. doi: 10.3390/biom16040586 (PMC13114193; doi:10.3390/biom16040586)

## Supplementary materials:

(a) RGD-binding site (site 1)

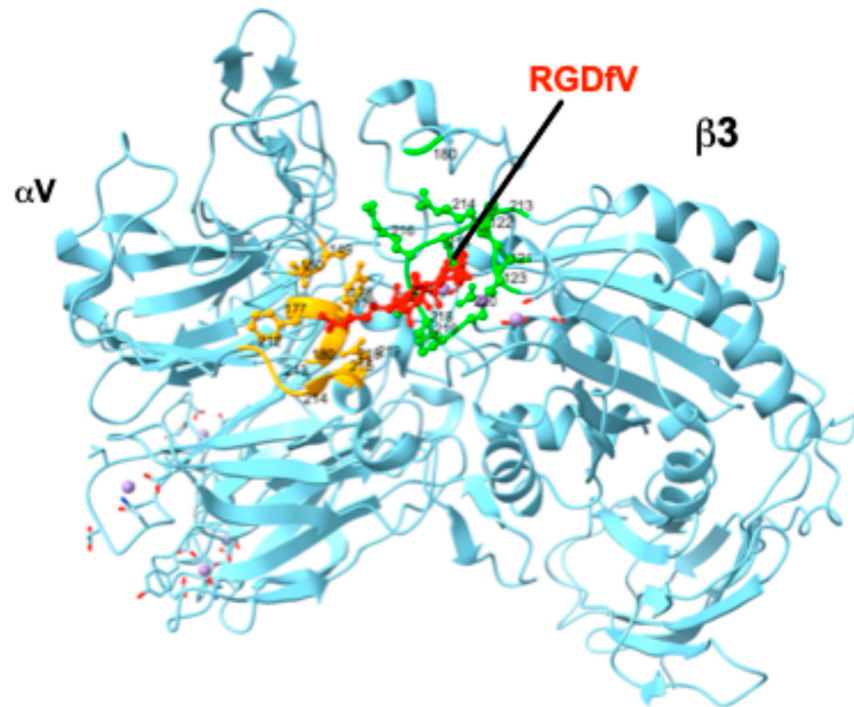

(b) Allosteric binding site (site 2)

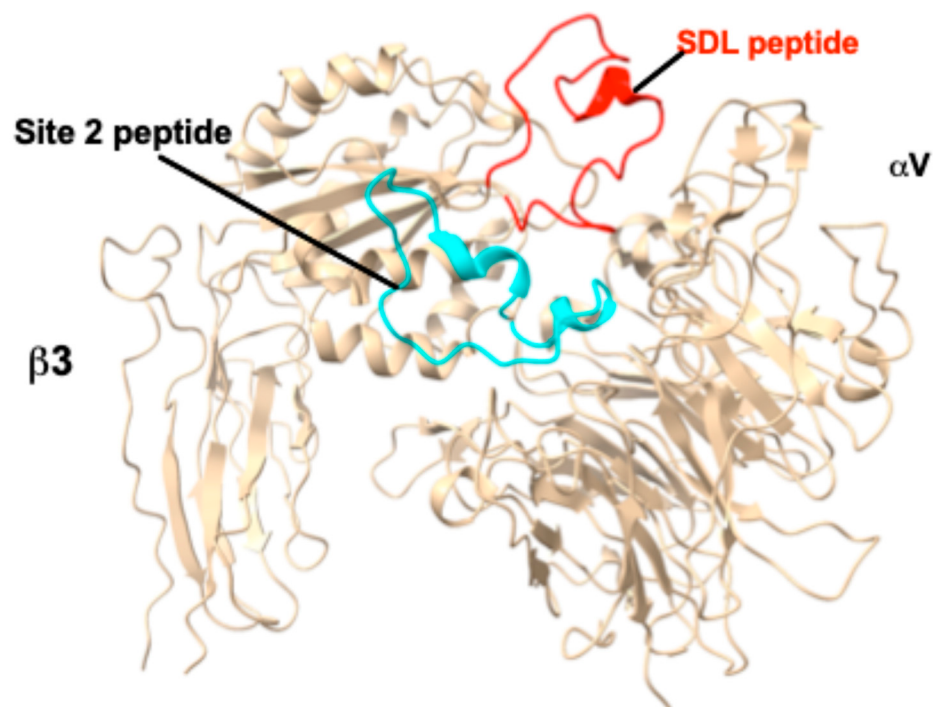

Supplement: Supplementary file 1 [file biomolecules-16-00586-s001.zip › biomolecules-4175625-supplementary.pdf]
